# An Analysis of Dr. Jackson's Work on Idiopathic Fever

**Published:** 1799-05

**Authors:** 


*Agreeably to the promise given in the last Number of this Journal, p.* 214, *we proceed to present our Readers with an Analysis of Dr.* JACKSON'S *interesting work on Idiopathic Fever, or "An Outline of the History and Cure of Fever, endemic and contagious," &c.* 8vo. 7s. Longman, 1798.

FROM the first dawnings of medical science, idiopathic fever has engaged the uniform attention of authors and practitioners. The importance of the subject sufficiently explains and justifies this circumstance. We might therefore, at this day, naturally conclude, that no subject connected with the practice of physic, would be so well understood as that of fever. The reverse of this, unfortunately, may be considered as the fact. Among other important causes of the deficiency of our knowledge on this subject, may be enumerated, the local situation of the most celebrated medical authors. Fever is the natural product of hot climates; whereas, the principal, if not the only authors of medical sects and systems, have, till lately, been limited to the temperate or cold regions of the northern hemisphere. Commercial advantages, and the wars undertaken to extend and protect them, have within the last twenty years, afforded opportunities to men of abilities and observation, of comparing the symptoms and progress of the same diseases, under the most diversified circumstances. This comparison has been productive of new facts, and new views of the subject, and thence of many considerable practical improvements. Whenever an important branch of science, which has continued stationary in the hands of the most celebrated teachers, for two or three hundred years, is attempted to be materially improved, the success will very much depend upon the manner in which, and by whom, the improvements are introduced. To succeed rapidly against colleges and established professors, it is necessary that the innovator should have all the personal advantages of education and talents, and all the professional ones of experience, united to a happy facility of explaining the new, without a supercilious contempt for the old, opinions. Such a character is Dr. Jackson. It would, however, be great injustice to several of his cotemporaries, to insinuate that he is the only labourer in this vineyard—ROBERTSON, TROTTER, CURRIE, and others of our own country, not to mention several in America and the East Indies, have contributed greatly to the same end. It will be unnecessary for us to mention the diligence, the talents for observation and description, the preparatory education, or means of information, which Dr. Jackson possessed: these will appear from his works. In the advertisement prefixed to the present volume, Dr. Jackson informs his readers, that he went out to Jamaica in 1774.

"Among the diseases of that country, *fever* was the one which presented itself the most frequently; as the most frequent, it seemed also to be the most important, and consequently occupied my chief attention. With the information acquired in this situation, and some dawnings of science in my mind, I went to North America, in 1778, partly with the view of prosecuting the study of fever in a new country, partly with the view of witnessing military service. Soon after my arrival in America, I was attached to the late 71st regiment; and in the various services of that corps, had the opportunity of observing very extensively, the different forms, and different degrees of fever, in the southern states of the American continent. I followed, in this country, the rule which I had observed in Jamaica, of writing down histories, and of arranging facts, at certain intervals. The result of this experience was given to the public in the year 1791.

"The views which I had obtained of fevers, by the mode of observation mentioned above, appeared in some respects different from the views of the medical writers, or practitioners with whom I had any acquaintance; but still something remained, which seemed to require confirmation. Under this impression, I offered service in the commencement of the present war, and was placed as surgeon to the third regiment of foot, or buffs, at that time embarked for the West Indies. The destination of this corps was changed on the eve of sailing: a circumstance which I then considered as a great disappointment; but as by these means an opportunity was furnished me of extending my views to the subject of *contagious fever*, the event has turned out to be fortunate---to myself, at least, it is satisfactory.

"In the year 1795, I was ordered to the West Indies, to the island of St. Domingo; where the duty assigned to me, afforded the means of examining the appearance of things at different parts, and in different districts, more fully than happened to any other person on the medical staff of that island. My former rule of conducting observations was adopted on this recent service; and the result, without reference to former observations or opinions, is now offered to the public, arranged after as clear an order as I have been capable of devising.

"The investigation of truth, as it concerns the health of man, has been the leading pursuit of my life; and this, perhaps, is my last offering to the public on the subject. I am aware that several of the opinions advanced here are heterodox; I am even aware that the manner in which they are sometimes brought forward, will be thought abrupt, if if not presumptuous; but if true, they may in time emerge in useful application: if otherwise, their progress, and consequent injury, will be small, for they do not enter the world under the patronage of a great name,

"ROBERT JACKSON."

We have given considerable extracts from this advertisement, that the public may judge for themselves, what may be expected from such a writer, who has enjoyed such opportunities for observation and experience. It ought to be observed in this place, that Dr. ROBERTSON enjoyed similar opportunities *; and, as he also appears to have devoted himself to the subject, for many years, and is no less determined to adopt nothing but what was supported by his own observation †; our readers will doubtless be pleased with a few occasional comparisons between the opinions of these two authors.

Dr. Jackson has divided his work into two parts; the first of which contains his opinions and observations on fever: the second may be considered as an appendix, on the principles of military discipline, &c. which does not occupy more than fifty pages. The first part is introduced by a short history of the origin and progress of contagious fever, as it appeared in different divisions of the British army in Holland, Ireland, and England, during part of the year 1793, the years 1794, 1795, and part of 1796. The first chapter Dr. Jackson commences with details of the situation of the troops, and the prevailing diseases while in the channel, and at Jersey. These details are interspersed with several valuable observations on the introduction of contagion into regiments and ships; with the means of preventing its spreading. We are next carried with the troops into Holland, where the various symptoms which presented themselves among the sick, are always described in pointed and impressive terms. In September, on the right bank of the Maese, where upwards of sixty men were attacked with fever within the space of twenty-four hours, he says,

"The symptoms of the complaint were violent in the commencement; the head-ach attacked suddenly, and severely distressed the patient; the eyes were often hot, painful, and turgid; the countenance flushed, cloudy, and grim; the limbs ached grievously, similar to the achings in the cold stage of intermittents, or accompanied with sensations of gnawing or tearing, more particularly along the shoulders and arms; the skin was generally dry, tender of the touch, or did not bear pressure without

* See an Essay on Fevers, wherein their theoretic genera, species, and various denominations, are, from observation and experience for thirty years, in Europe, Africa, and America, and on the intermediate seas, reduced under their characteristic genus *Febrile infection;* and the cure established on philosophical induction. By ROBERT ROBERTSON, M.D. 8vo. Robinson, 1790.

† See pages 3. 86. 168, &c.

without pain; the heat was often ardent, and sometimes pungent; the pulse was insidious, sometimes agitated and irregular, sometimes apparently little disordered in point of time, but seldom energetic, elastic, and expanding: the tongue was usually *white and slimy*; to which was often added nausea, and sometimes vomiting: thirst was irregular; rest was altogether wanting, or the sleep was disturbed by dreams; the state of the bowels was uncertain; costiveness prevailed, or a purging approached to dysentery: in many instances, there were convulsive or spasmodic motions of the moving powers, tremors, startings, affection of the organs of respiration, alternating with affection of the head. This disease did not terminate in regular intermissions, but it was disposed to subside in three or five days, it seldom extended to seven. It often terminated finally in a form of dysentery, or in local disease of an organ".—p. 13.

After the troops were withdrawn from the continent, in May, 1795, the attention of Government seems to have been directed towards the French islands in the West-Indies. With this view, two expeditions were formed, the one directed against the Charibean islands, the other, destined for St. Domingo, assembled at the Cove of Cork, and was chiefly composed of drafts from newly raised regiments serving in Ireland. In this the author occupied a situation on the medical staff. To prevent desertion and irregularity among the troops, which appeared in September, an encampment was formed on the island of Spike, an unsheltered island in the harbour of Cove. There the seeds of fever sprung up, and by the time the troops were all embarked, the disease was aggravated to an uncommon degree of virulence.

"From the middle of October to the middle of February, not fewer than 500 men (out of 9000) were numbered with the dead; during which period, not fewer than 3000 had been mustered on the list of sick. The causes are obvious, and similar to those which produced such ravages on the continent, viz. the seeds of infection, incautiously introduced into the army by the recruits of independent companies, called into activity by a variety of causes, concentrated and exalted into a degree of pestilential virulence, by accumulation in a narrow space."—p. 35.

Dr. Jackson, after stating the various symptoms that occurred in the different degrees of fevers which harrassed these forces, adds,

"The sickness which prevailed among the troops assembled at the Cove of Cork, for the reinforcement of St. Domingo, furnishes an extensive field of observation on the nature of the contagious fever of armies. The operation of the cause manifested itself in a great variety of forms. It was evidently connected with *sores, and spreading ulcers of the legs*, and more evidently still, with diarrhæa, with dysentery, or severe gripings and bloody evacuations: the irregular forms, and slighter degrees of fever, were numerous; the violent and threatening, occurred daily; and the concentrated, suspending life, as it were, by a direct operation, appeared on different occasions. While the troops were on shore under tents, in wet and stormy weather, the prevailing form was dysenteric; it often became febrile under a roof, or on board the small vessels employed as hospitals; it generally became febrile after embarkation.

It may be further remarked, that the form soon became febrile, violent and concentrated, in the crouded hospital of the fort; that the mortality was great, and that life there seemed often to be suffocated or arrested without struggle or resistance. In the barns, hovels and sheds, the appearances were more irregular, and the symptoms were more threatening, but the mortality was comparatively small, and recoveries were frequently rapid. On board of transports, where the patients remained below during the greatest part of the time, the disease was violent, concentrated, and speedily fatal: where brought on deck, and remaining on deck during the day, the effects were similar to those in the sheds on shore. Under confinement between decks, the disease seemed to retain its full power of contagion; under exposure to the air on deck, this power was evidently weakened; and where to this exposure were joined daily washing of the body and purifying of the bedding, it was so far weakened, that its existence by the time of arrival in the West Indies, was in many cases doubtful. This disease follows a law essentially different from the *periodical movements* of endemic fevers." p. 44.

Dr. Jackſon next proceeds to examine more minutely, the means by which contagion is brought into and propagated among armies and navies. His obſervations on this ſubject, appear highly important, but we think them more particularly directed to the Commander in Chief, than to the medical board.

The troops being arrived in St. Domingo, the author employs his ſecond chapter in delineating the ſituations, ſoils, aſpects, winds, and elevations of the ſtations, occupied by the Britiſh troops, together with their effect on the health or modification of diſeaſe. As theſe particulars can be only intereſting to perſons going to the iſland, who will doubtleſs conſult the work itſelf, we ſhall give few extracts from this part. By a compariſon of the endemic fever which occurred in various ſituations of St. Domingo, with the contagious fever of Europe, the author draws the following inferences. The endemic diſeaſe of all the Weſt India Iſlands, is fever, diarrhœa, ſore legs, or ſpreading ulcers of a peculiar kind. Theſe complaints moreover appear ſo connected with each other, as to afford a preſumption that they depend fundamentally upon the ſame general cauſe, differing in degree of force, or differently modified according to circumſtances of ſeaſon, place, and ſituation. The connection alluded to is obvious in the endemic, and a ſimilar connection is obſerveable under the action of the contagion of jails, hoſpitals, crouded barracks, and crouded ſhips.

It appears from a peruſal of their hiſtories, that nearly all the iſlands of the Weſt Indies are under ſimilar laws, with regard to health. In towns and places near the ſea coaſts, European ſoldiers ſicken and die; in the mountainous and interior parts of the country, health ſuffers little, and mortality is inconſiderable. The ſubſequent obſervations in this chapter being of a political nature, we paſs them over.

The

The author deriving his information from fources fuch as we have ftated, advances from the enumeration of facts, to the formation of doctrines; and, in his third chapter, enters upon the CAUSES of Idiopathic fever. This part of the fubject is fo important, and as it forms the bafis of his twofold diftinctions of fevers, viz. endemic and contagious, we conclude that our readers would be much difappointed, if we did not lay before them the authors reafoning upon it.

T. B.

(To be continued in our next.)

